# Expression of epithelial calcium transport system in rat cochlea and vestibular labyrinth

**DOI:** 10.1186/1472-6793-10-1

**Published:** 2010-01-29

**Authors:** Daisuke Yamauchi, Kazuhiro Nakaya, Nithya N Raveendran, Donald G Harbidge, Ruchira Singh, Philine Wangemann, Daniel C Marcus

**Affiliations:** 1 Cellular Biophysics Laboratory, Dept. Anatomy & Physiology, Kansas State University, Manhattan, KS 66506, USA; 2 Cell Physiology Laboratory, Dept. Anatomy & Physiology, Kansas State University, Manhattan, KS 66506, USA; 3 Dept. of Otolaryngology-Head and Neck Surgery, Tohoku University Graduate School of Medicine, Sendai 980-8574, Japan

## Abstract

**Background:**

The low luminal Ca^2+ ^concentration of mammalian endolymph in the inner ear is required for normal hearing and balance. We recently reported the expression of mRNA for a Ca^2+^-absorptive transport system in primary cultures of semicircular canal duct (SCCD) epithelium.

**Results:**

We now identify this system in native vestibular and cochlear tissues by qRT-PCR, immunoblots and confocal immunolocalization. Transcripts were found and quantified for several isoforms of epithelial calcium channels (TRPV5, TRPV6), calcium buffer proteins (calbindin-D9K, calbindin-D28K), sodium-calcium exchangers (NCX1, NCX2, NCX3) and plasma membrane Ca^2+^-ATPase (PMCA1, PMCA2, PMCA3, and PMCA4) in native SCCD, cochlear lateral wall (LW) and stria vascularis (SV) of adult rat as well as Ca^2+ ^channels in neonatal SCCD. All components were expressed except TRPV6 in SV and PMCA2 in SCCD. 1,25-(OH)_2_vitamin D_3 _(VitD) significantly up-regulated transcripts of TRPV5 in SCCD, calbindin-D9K in SCCD and LW, NCX2 in LW, while PMCA4 in SCCD and PMCA3 in LW were down-regulated. The expression of TRPV5 relative to TRPV6 was in the sequence SV > Neonatal SCCD > Adult SCCD > LW > primary culture SCCD. Expression of TRPV5 protein from primary culture of SCCD did not increase significantly when cells were incubated with VitD (1.2 times control; P > 0.05). Immunolocalization showed the distribution of TRPV5 and TRPV6. TRPV5 was found near the apical membrane of strial marginal cells and both TRPV5 and TRPV6 in outer and inner sulcus cells of the cochlea and in the SCCD of the vestibular system.

**Conclusions:**

These findings demonstrate for the first time the expression of a complete Ca^2+ ^absorptive system in native cochlear and vestibular tissues. Regulation by vitamin D remains equivocal since the results support the regulation of this system at the transcript level but evidence for control of the TRPV5 channel protein was lacking.

## Background

The calcium concentration of the inner ear luminal fluid, endolymph (cochlea 23 μM and vestibule 280 μM), is much lower than that of the basolateral fluid, perilymph (ca. 1 mM) [1]. The low Ca^2+ ^level is essential for the normal transduction of sound and acceleration for hearing and balance [2]. It has been assumed that there likely are one or more Ca^2+ ^absorptive mechanisms in the inner ear. We previously demonstrated that transcripts of a novel Ca^2+ ^absorptive system are expressed in primary cultures of rat semicircular canal duct (SCCD).

The Ca^2+ ^absorptive system [3] is comprised of an apical membrane entry pathway (the Ca^2+^-selective TRPV5 and/or TRPV6 channel), a cytosolic Ca^2+ ^buffer protein (calbindin-D9K and calbindin-D28K), and basolateral Ca^2+ ^exit pathways (sodium-calcium exchangers (NCX) and plasma membrane Ca^2+^-ATPases (PMCA)). Expression of these transporters can be regulated by the active form of vitamin D, 1,25-(OH)_2_vitamin D_3 _[4]. Ca^2+ ^that enters the cell through the apical TRPV5/6 channel is buffered by the calbindin and transported by diffusion in bound form across the cell, from which it is released across the basolateral cell membrane via the PMCA and NCX transporters. The predominant apical channel in kidney is TRPV5 and in the intestine it is TRPV6 [4].

Observations of this transport system in the ear have been limited to transcript expression in primary cultures of rat SCCD for all of the transport system genes [5], immunolocalization in mouse cochlea and vestibular labyrinth of TRPV5 and TRPV6 [6], and radiolabeled Ca^2+ ^fluxes in primary cultures of rat SCCD [7].

The questions remained whether 1) the system of epithelial Ca^2+ ^transport genes was also expressed in the cochlea, 2) whether native inner ear tissues expressed the same constellation of genes as the primary cultures of SCCD, 3) whether transcript expression in rat tissues resulted in protein expression of epithelial Ca^2+ ^channel genes with a distribution similar to mouse and 3) whether tissues from each represented region of the inner ear that express epithelial Ca^2+ ^transport genes responded to 1,25-(OH)_2_vitamin D_3_. The present study addressed those questions. Evaluation of protein expression used primary cultures to obtain sufficient material for immunoblots and used sections of rat cochlea and SCCD for immunostaining.

## Results

The "primary cultures" of SCCD refer to epithelial cells from neonatal SCCD that have been seeded on permeable supports and have proliferated to confluence over 5 or more days. The proliferation and re-differentiation *in vitro *and the attachment to a new surface all carry the risk of altered gene expression compared to canals *in vivo*. The tissues that we refer to as "native" were explants that were incubated for only 24 hours *in vitro*, that experienced no changes to proliferative and re-differentiation phases nor attachment to a new surface. The acute incubation of the explants was necessary to study possible changes to gene expression in response to vitamin D and for an untreated control series.

### mRNA expression

**Figure 1:**
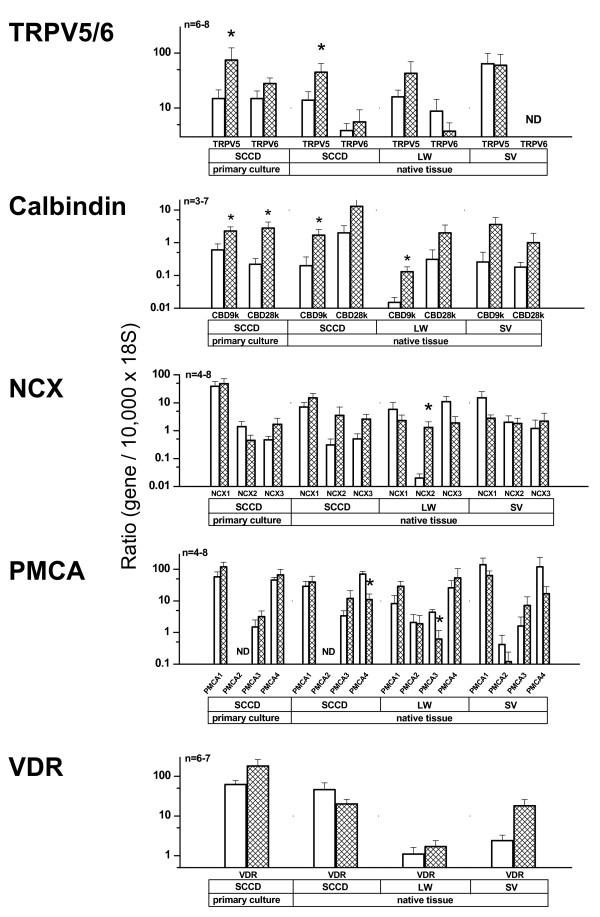
**Summary of expression of transcripts for isoforms of epithelial Ca^2+ ^channels (TRPV5 and TRPV6), calbindin, NCX, PMCA and VDR with and without exposure to 1,25- (OH)_2_vitamin D_3_**. Expression levels given as ratio of specific gene to 18S in primary cultures of semicircular canal duct (SCCD), native SCCD, native lateral wall (LW) and native stria vascularis (SV). All native tissues were incubated overnight after microdissection in order to reduce extracellular cross-contamination from RNA released from adjacent tissues (see Fig 1B). Open bars; control, hatched bars; 1,25-(OH)_2_vitamin D_3 _treated, Significant up- or down-regulation is indicated; *, P < 0.05; ND, not detected. For comparison, data in the left section are primary culture of SCCD, reproduced from [5].

Levels of transcript expression for all components and isoforms of the epithelial calcium absorption pathway are shown in Fig. 1 for isolated native tissues from the vestibular labyrinth (semicircular canal; SCCD) and cochlea (stria vascularis and lateral wall exclusive of stria). Results from primary cultures of neonatal SCCD [5] are shown for reference.

The epithelial calcium channel TRPV5 was expressed in all tissue fractions, while TRPV6 was expressed in all tissue fractions except stria vascularis. The absolute quantities of each gene transcript cannot be unambiguously compared since the efficiency of the RT step for each primer is not known. However, the ratios between the apparent TRPV5 and TRPV6 transcripts can be compared among tissues. The apparent TRPV5:TRPV6 transcript expression ratio in *adult *SCCD was 3.2, *neonatal *SCCD was16, much higher in stria vascularis since TRPV6 was not detectable but only 1.8 in the lateral wall. The high TRPV5:TRPV6 ratio in *adult *SCCD compared to *neonatal *primary cultures ([5] and Fig. 1) was not a result of changes during development. TRPV5 in native *neonatal *SCCD was expressed at 16 times the level of TRPV6 (TRPV5 = 4.7 × 10^-3^, n = 12; TRPV6 = 2.9 × 10^-4^, n = 10; normalized to 10,000 18S). The expression of TRPV5 relative to TRPV6 is therefore in the sequence Stria vascularis > Neonatal SCCD > Adult SCCD > cochlear lateral wall > primary culture SCCD.

The calcium-buffering calbindin proteins, calbindin-D9K and calbindin-D28K, were expressed in native SCCD, lateral wall and stria vascularis of adult rat as well as in primary culture SCCD (Fig. 1). The relatively low gene expression levels reported here likely reflects a low reverse transcription efficiency of the primers but may also reflect a low level of transcript expression. All isoforms of the sodium-calcium exchanger (NCX1, NCX2 and NCX3) were expressed as mRNA by all native tissues as well as by cultured SCCD (Fig. 1). All four isoforms of the plasma membrane calcium ATPase (PMCA) were found in the cochlear tissues, while only three isoforms (not PMCA2) were found in the SCCD (native and cultured) (Fig. 1). The relatively low level of expression of PMCA2 in stria vascularis suggests a minor role in the function of this tissue and could conceivably represent a low level of contamination from other tissues. Vitamin D receptor transcripts were found in all native tissues (Fig. 1) as well as in primary cultures of SCCD.

Addition of 1,25-(OH)_2_vitamin D_3 _for 24 h increased the expression of TRPV5 in native SCCD (× 3.2), as in primary cultures [5], but not significantly in lateral wall or stria vascularis (Fig. 1). Calbindin-D9K was significantly up regulated by 1,25-(OH)_2_vitamin D_3 _in native SCCD (× 3.4) and lateral wall (× 8.9) but not in stria vascularis (× 13.9; P = 0.059). 1,25-(OH)_2_vitamin D_3 _did not significantly up-regulate the expression of calbindin-D28K in any of the native tissues, even though it did in primary cultures of SCCD (Fig. 1). NCX2 in the lateral wall was the only sodium-calcium exchanger that was up-regulated (× 101) by 1,25-(OH)_2_vitamin D_3_. None of the PMCAs were up-regulated by 1,25-(OH)_2_vitamin D_3_, while there were small but significant decreases of PMCA4 in SCCD and of PMCA3 in lateral wall. 1,25-(OH)_2_vitamin D_3 _exposure did not significantly change the expression level of the vitamin D receptor in any tissues.

### Protein expression of TRPV5: immunoblot

**Figure 2:**
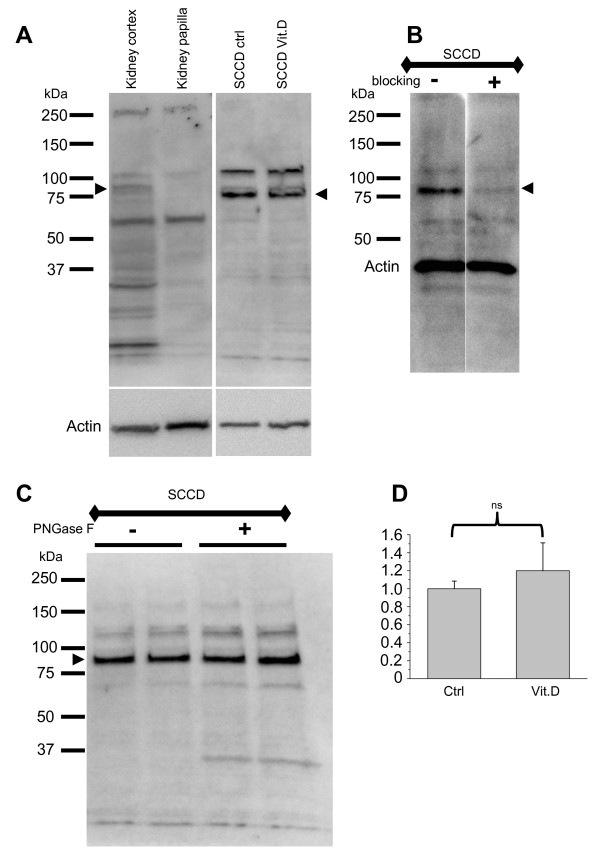
**TRPV5 protein detected by immunoblot**. Incubation of primary cultures of SCCD for 24 h in the absence and presence of 100 nM 1,25- (OH)_2_vitamin D_3_. Protein expression of TRPV5 was compared with β-actin. *A) *Cultured SCCD cells with or without 1,25-(OH)_2_vitamin D_3_; kidney cortex and papilla were used for positive and negative controls. The blot was stripped of TRPV5 antibody and re-probed for beta-actin. *B) *Antigenic peptide blocked 80 kDa band. A band at 42 kDa is β-actin. The blot was directly probed for beta-actin without stripping since the TRPV5 antibody did not produce bands at the molecular weight of actin. *C) *PNGase F did not affect any TRPV5 bands. A band at 36 kDa is PNGase F itself. *D) *Intensity of 80 kDa TRPV5 bands standardized to β-actin. The mean intensity in 1,25-(OH)_2_vitamin D_3 _treated cells was larger but not statistically significant. Ctrl, control; Vit.D, 1,25-(OH)_2_vitamin D_3 _; ns, P > 0.05; n = 14 each condition.

TRPV5 protein migrated to about 80 kDa (linear interpolation) for cultured SCCD as well as native kidney cortex (positive control) of neonatal rat but not in kidney papilla (negative control; (Fig. 2A). Pretreatment of the primary antibody with antigenic peptide (Fig. 2B) markedly reduced the intensity of the 80 kDa band, whereas the band near 120 kDa was not blocked with antigenic peptide. Non-specific bands appeared sporadically at 65 kDa in kidney and SCCD. The suggestion of a double band (80 kDa and ~100 - 120 kDa) in SCCD could represent two types of post-translational modification, two levels of glycosylation or non-specific labelling of the upper band. The 80 kDa band is similar in size to the calculated molecular weight of TRPV5, 82.4 kDa. Exposure to 1,25-(OH)_2_vitamin D_3 _did not significantly increase protein expression of TRPV5 in the primary cultured cells. Xenopus leavis oocytes transfected with human TRPV5 cDNA showed 2 additional bands for TRPV5 and they were sensitive to PNGase F [8]. By contrast, there were no bands from SCCD that were susceptible to PNGase F treatment (Fig. 2C). The antibody for TRPV6 did not react with specific bands in immunoblots.

### Protein expression of TRPV5 and TRPV6: immunohistochemistry

**Figure 3:**
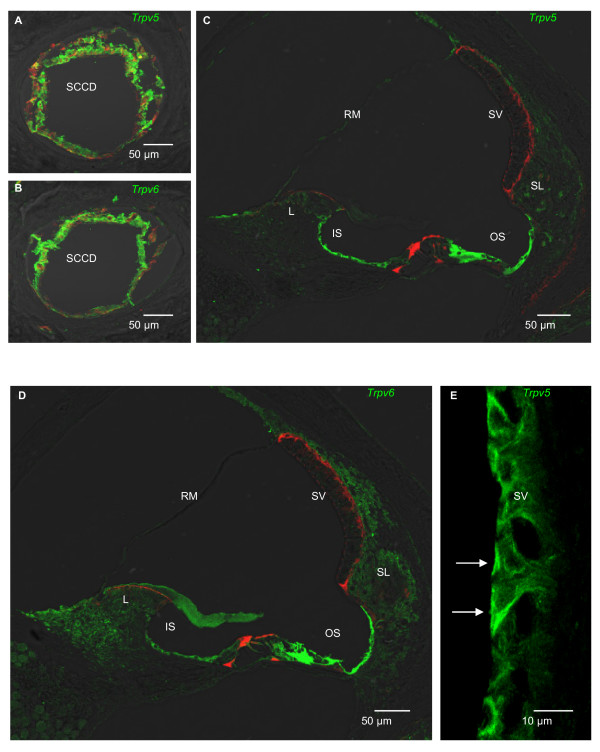
**Epithelial calcium channel immunolocalization in the cochlea and vestibular system of rat**. Double-staining with an antibody against TRPV5 or TRPV6 (green) and actin (red; phalloidin) (A-D) and single-staining with an antibody against TRPV5 (green) (E). A, B) Cross sections of SCCD were stained against TRPV5 and TRPV6. C) Cross section of cochlea was stained against TRPV5. Outer sulcus cells (OS), inner sulcus cells (IS) and Hensen's cells (left of OS) show significant staining for TRPV5. D) Cross section of cochlea was stained against TRPV6. Outer sulcus cells, inner sulcus cells and Hensen's cells show significant staining for TRPV6. E) Cross section of stria vascularis (SV). Marginal cells of stria vascularis (arrows) show staining for TRPV5 at the apical membrane. Sections exposed to primary antibodies preabsorbed with antigenic peptide were not stained (not shown). RM, Reissner's membrane; L, spiral limbus; SL, spiral ligament.

The distribution of the epithelial calcium absorptive system was examined in more detail by immunostaining of cochlear and vestibular structures for TRPV5 and TRPV6 (Fig. 3). In the vestibular system (Fig. 3A,B), the only staining of epithelial cells was observed in the SCCD. The SCCD epithelial cells are only 1 μm tall, making it difficult to clearly discern differential staining of the apical and basolateral membrane for low copy number membrane proteins. We visualized specific labeling of TRPV5 and TRPV6 in the SCCD by opening the pin-hole of the confocal microscope in order to integrate the signal over a greater area and depth (Fig. 3A,B). The specificity of the signal was assured by the total absence of staining of the surrounding bone and of sections prepared in the absence of primary antibody (not shown). No epithelial staining was seen in the utricle or ampullae (not shown). Phalloidin staining aided the identification of epithelial cells by the location of the actin ring associated with the tight junction complex.

In the cochlea (Fig. 3C,D,E), strong immunostaining for TRPV5 and TRPV6 was observed in both the inner sulcus and outer sulcus epithelial cells, as well as the Hensen's cells medial to the outer sulcus (Fig. 3C,D). The outer sulcus is part of the "lateral wall" fraction included in the mRNA determinations. The observations of TRPV5 and TRPV6 protein expression in the inner sulcus and Hensen's cells extends our knowledge of the expression pattern. Marginal cells of the stria vascularis were labeled for TRPV5 (Fig. 3E) but not TRPV6 (not shown) protein expression near the apical membrane. The staining in stria vascularis for TRPV5 was repeatable but variable. When the stria vascularis was immunoreactive, the marginal cells were strongly stained (Fig. 3E).

## Discussion

The level of endolymphatic [Ca^2+^] is controlled by both secretory mechanisms (e.g., PMCA2 in the apical surface of hair cells [9]) and absorptive mechanisms. The present paper reports the first evidence for the expression of all genes necessary to constitute a complete transepithelial Ca^2+ ^absorptive pathway in native tissues of the inner ear. We previously reported that cochlear outer sulcus cells and vestibular transitional cells have apical nonselective cation channels that are likely permeable to Ca^2+ ^[10,11], but their contribution to transepithelial Ca^2+ ^absorption is not known.

### Expression

The transmural absorption of Ca^2+ ^by a number of epithelia has recently been ascribed to a set of genes that encode the apical entry channels TRPV5 and TRPV6, the cytosolic Ca^2+ ^buffering proteins calbindin-D9K and calbindin-D28K, and the basolateral Ca^2+^-extruding transporters sodium-calcium exchangers (NCX) and plasma membrane calcium ATPases (PMCA) [3]. In other epithelial, either TRPV5 or TRPV6 is expressed at a higher level, but in spite of the large array of epithelia surveyed previously [3], expression of this transport system in the inner ear was not reported. We demonstrated here that all components of the epithelial Ca^2+ ^channel transport system were found to be expressed as transcripts in the vestibular SCCD and cochlea.

Our results validate the primary culture of SCCD in terms of the presence of all the genes that participate in Ca^2+ ^absorption and regulation, although the relative expression level of TRPV5 and TRPV6 was observed to be substantially reduced in culture compared to the native cells. This apparent discrepancy is consistent with the lack of 1,25-(OH)_2_vitamin D_3 _in the control culture medium (see below), whereas the native tissues were exposed to intermediate levels of the hormone *in vivo *prior to sacrifice. TRPV5 and TRPV6 form homo- and hetero-tetrameric channel complexes and regulation of the relative expression levels of TRPV5 and TRPV6 may be a mechanism for fine-tuning Ca^2+ ^transport kinetics in TRPV5/6-expressing tissues [12].

Quantification of expression levels has intrinsic problems. First, there can be large discrepancies between levels of transcripts and levels of the corresponding proteins. Further, the proteins can receive various post-translational modifications and trafficked to different destinations with a consequent multiplicity and altered level of function. Second, seemingly anomalous results due to imprecision of Ct determination in RT-PCR and its logarithmic relation to transcript number can occur, leading to calculated large fold-changes in expression that do not reach statistical significance (e.g., calbindin-D9K in stria vascularis; *vide infra*).

TRPV5 and TRPV6 gene expression in the inner ear was also demonstrated at the level of protein. Sufficient protein could be obtained from primary cultures of SCCD to allow immunoblot observation of TRPV5. Interestingly, immunoblot data did not show a significant difference in TRPV5 expression between control and 1,25-(OH)_2_vitamin D_3 _treated SCCD cells. This result is not consistent with the functional data from the same preparation that show an upregulation by 1,25-(OH)_2_vitamin D_3 _of radiolabeled Ca^2+ ^fluxes in primary cultures of SCCD [7]. The apparent discrepancy could be accounted for by 1) the increased transcript expression not affecting protein amount and 2) an upregulation by 1,25-(OH)_2_vitamin D_3 _of another controlling part of the transport system. The ineffectiveness of PNGase F treatment for TRPV5 suggests the occurrence of other posttranslational modifications than glycosylation or a low abundance of glycosylated protein.

Immunolocalization of both epithelial Ca^2+ ^channels was consistent with the transcript data for both the vestibular system and for the cochlea. In addition to the cochlear localization of the strial marginal cells and the outer sulcus of the lateral wall, these channels were also observed in the inner sulcus epithelial cells of the rat inner ear. Recent observations in mouse show similar cellular locations, although the preponderance of TRPV5 or TRPV6 in some cells differs [6]. Our data demonstrate that the earlier findings were not strictly limited to one species. The antibodies used do not restrict their binding to membrane proteins, so that there is staining of protein in the cytosol, preventing the subcellular localization of TRPV5 and TRPV6 to the apical and/or basolateral membranes. In addition, the antibody against TRPV6 did not function in immunoblots.

The other genes required for Ca^2+ ^absorption--calbindin-D9K, calbindin-D28K, NCX1 and PMCA1b--were previously found to be involved in Ca^2+ ^absorption by the kidney [13,14]. Calbindin-D9K can prevent calcium-dependent inactivation of TRPV5/6 by buffering overloaded Ca^2+ ^beneath the apical side while calbindin-D28K is freely diffusible in the cytoplasm [4]. We evaluated all isoforms of NCX1-3 and PMCA1-4 in the inner ear tissues.

PMCA was demonstrated by immunohistochemistry to be present in the basolateral membrane of strial marginal cells and Reissner's membrane epithelial cells [15]. NCX1 and PMCA1 were the predominant isoforms expressed in the inner ear, as in the kidney. However, other isoforms were also found to be present. PMCA2 was not expressed in either native or cultured SCCD and virtually absent in stria vascularis. Absence of PMCA2 in stria vascularis is compatible with previous reports [9,16]. Expression of PMCA2 in the lateral wall fraction might arise from contributions by Reissner's membrane [16]. PMCA2 is therefore not likely important for inner ear Ca^2+ ^absorption; rather its function is likely limited to Ca^2+ ^secretion at the apical membrane of hair cells [9,17] and possibly Reissner's membrane [16].

Our detection of PMCA3 and PMCA4 in all tissue fractions is at variance with previous reports for the cochlea [9,16]. It is possible to attribute these discrepancies to species differences. A previous immunohistochemical study showed calbindin-D28k expression in musk shrew spiral ganglion, inner and outer hair cells, but expression in lateral wall was only observed during pre-natal ages [18]. By contrast, we observed expression of calbindin-D28k in lateral wall fractions of young adult rats.

### Regulation

A wide range of signal pathways converge to control Ca^2+ ^absorption. An important control mechanism is regulation of the number of expressed apical entry Ca^2+ ^channels and calbindins by 1,25-(OH)_2_vitamin D_3_. It is apparently characteristic of different epithelia that 1,25-(OH)_2_vitamin D_3 _up-regulates predominantly one channel isoform through activation of the vitamin D receptor [3]. Caco-2 cells up-regulate TRPV6 in a time- and dose-dependent manner [19]. By contrast, 1,25-(OH)_2_vitamin D_3 _up-regulates TRPV5, but not TRPV6, in kidney [3] and SCCD (*vide infra*).

Two isoforms of calbindin, calbindin-D9K and -D28K, are expressed in the inner ear epithelia and calbindin-D9K transcript expression is up-regulated by 1,25-(OH)_2_vitamin D_3_. It has been shown that calbindin-D9K is localized near both the apical and basolateral plasma membranes [20], while calbindin-D28K is freely diffusible in the cytoplasm [20] but translocates to the apical membrane to associate with the TRPV5 [21]. One function of calbindin-D28K is to prevent Ca^2+ ^from binding to calmodulin, which can block TRPV6 by binding to the COOH-terminal region [22]. Interaction with either channel is important since the functional channel is likely a tetraheteromer of TRPV5/TRPV6. Both calbindins, NCX1 and PMCA1b genes were up-regulated in hormone-deficient 1α-OHase^-/- ^mice by 1,25-(OH)_2_vitamin D_3 _[20].

NCX and PMCA isoform expression levels were not uniformly changed by 1,25-(OH)_2_vitamin D_3 _in the SCCD, lateral wall and stria vascularis. NCX2 in the lateral wall may be important for the Ca^2+ ^absorption pathway because of its up-regulation by 1,25-(OH)_2_vitamin D_3_. But the reason why PMCA4 in SCCD and PMCA3 in lateral wall were down-regulated is not clear. The lack of response of all genes in the stria vascularis may indicate that this transport system is not functional in stria. Alternatively, it may represent a vitamin D-insensitive transport system in this tissue.

The activity of the TRPV5 and TRPV6 channels is steeply controlled by both intra- and extracellular pH [23]. The strong inhibition by acid extracellular pH is of particular interest in view of pathologic conditions leading to lowered pH in both cochlear and vestibular endolymph (see below). Epithelial Ca^2+ ^channel activity can further be controlled by PIP2, providing a link to a variety of G protein-coupled receptors [24].

### Physiological significance

The question naturally arises whether this transport system is relevant at the whole organ level. Several observations demonstrate the functionality of this system and point to its importance in hearing and balance. It was recently shown that SCCD epithelial cells take up radiolabeled Ca^2+ ^more rapidly from the apical side than from the basolateral side, that the net uptake is increased in the presence of 1,25-(OH)_2_vitamin D_3 _and that the apical uptake is inhibited by acidic luminal pH (a hallmark of TRPV5 and TRPV6) [7]. It was therefore predicted that any condition that results in an acidification of endolymph would also lead to increased luminal [Ca^2+^] and a consequent decrease in cochlear and vestibular function, which depend on the normally low endolymphatic [Ca^2+^] [2]. Indeed, mutations or deletion of the bicarbonate-secreting transporter pendrin (expressed in the luminal membranes of cochlear and vestibular epithelial cells) lead to hearing and balance deficits in humans [25] and mice [26]. Deletion of pendrin led to acidified endolymph and consequently a dramatic elevation of endolymphatic [Ca^2+^] in both the cochlea [6] and the utricle [7]. This observation suggests that the TRPV5/6 Ca^2+ ^absorption system plays a highly significant physiological role in endolymph Ca^2+ ^homeostasis, even though the endocochlear potential apparently plays a strong role by providing a driving force for passive efflux [27].

Calcium homeostasis in the inner ear via TRPV5/6 could play an important role in causes of benign paroxysmal positional vertigo (BPPV) and may therefore be an effective drug target. BPPV is characterized by brief episodes of nystagmus and vertigo in response to certain movements of the head. It is thought to be caused by dislodged otoliths (composed primarily of CaCO_3 _crystals) from the utricle that enter one of the semicircular canals, leading to inappropriate stimulation of canal. A recent study reported that strong correlations in human patients were observed between diagnosis of BPPV and disturbed calcium homeostasis as reflected in reduced bone mineral density [28].

Vitamin D has been implicated in hearing function. Ikeda and associates found that vitamin D deficiency resulted in hearing impairment in rats [29] and that 80% of patients in a study with bilateral sensory neural hearing loss (BSNHL) were found to be deficient in 1,25-(OH)_2_vitamin D_3 _[30,31]. Importantly, the patients with BSNHL and low serum vitamin D had normal serum Ca^2+^, consistent with a local effect of vitamin D deficiency in the auditory and vestibular periphery. The vitamin D-deficient rats had a reduced perilymphatic [Ca^2+^] level, making the interpretation less clear. It was not known whether the observed effects were due to direct effects on the epithelial calcium channel system (not known at that time), to the lowered systemic [Ca^2+^], or to other causes. Nonetheless, the correlations are consistent with a direct action on the system reported here.

The dysfunction of Ca^2+ ^absorption by mutation or absence of the TRPV5 and TRPV6 genes would be expected to lead to impaired hearing and balance. It is perhaps more than a coincidence, therefore, that the genes for TRPV5 and TRPV6 are located on chromosome 7q35 and 7q33-34 respectively in human [4] and the locus of the non-syndromic deafness gene [32] DFNB13 is located at the encompassing region on chromosome 7q34-36 [33].

## Conclusions

Both the cochlea and the vestibular system were found to express all genes needed to constitute a Ca^2+ ^absorption mechanism that can maintain the low levels of endolymphatic [Ca^2+^] needed to sustain normal hearing and balance. The native semicircular canal expresses all of the Ca^2+ ^transport genes found previously in the culture system. No other epithelial cells in the vestibular system were found to express epithelial Ca^2+ ^channels. Specific cochlear epithelial cells that express TRPV5 and TRPV6 include those of the inner and outer sulcus and Hensen's cells. Strial marginal cells of the cochlea also express TRPV5 but not TRPV6. These components of the epithelial Ca^2+ ^absorption pathway likely play an important role in Ca^2+ ^homeostasis of endolymph.

## Methods

### Tissue isolation and incubation

Temporal bones were obtained from adult (3-5 weeks) or neonatal (3-8 d) Wistar rats according to a protocol approved by the Kansas State University Institutional Animal Care and Use Committee. SCCD were microdissected from the bony canal, excluding the common crus. Cochleae were microdissected and stria vascularis tissue was isolated by peeling carefully from spiral ligament under microscopic control. The rest of the lateral wall, including spiral ligament, Reissner's membrane, spiral prominence and outer sulcus cells was collected as "lateral wall".

Isolated tissues from adult animals and dispersed SCCD epithelial cells from neonatal animals were incubated on permeable supports in DMEM/F-12 medium (Invitrogen 12500-062 Carlsbad, CA), 5% fetal bovine serum, 100 U/ml penicillin, and 100 μg/ml streptomycin) in 5% CO2 atmosphere at 37°C as described previously [34]. Culture inserts were 6.5 mm diameter Transwells (Costar #3470, Corning, NY) for RNA isolation and 12 mm Snapwell inserts (Costar #3801) for protein isolation.

Isolated intact tissues did not proliferate in acute (24 h) culture but dispersed SCCD cells proliferated rapidly to confluence (primary cell culture), typically within about 5 days. Intact explants of isolated tissues were incubated (24 h) in the presence or absence of 1,25-(OH)_2_vitamin D_3 _(100 nM DM-200; BIOMOL, Plymouth Meeting, PA; dissolved in 0.1% ethanol); incubation with 0.1% ethanol was used as control.

### RNA Isolation

Total RNA was extracted from the tissue explants or SCCD primary culture cells using QIAshredder Mini Spin Column (79654, Qiagen, Valencia, CA) and RNeasy Micro Kit following the manufacturer's protocol (#74004, Qiagen, Valencia, CA). Total RNA quality was determined with an Agilent BioAnalyzer (Model 2100, Palo Alto, CA) with RNA 6000 Nano/Pico Assay Kits (5065-4473/5065-4476, Agilent technology) (See Additional File 1: Fig. S1) and quantity was determined with a Nanodrop ND-1000 Spectrophotometer (NanoDrop Technologies, Delaware, USA), using a conversion factor of 1 absorption unit normalized to 1.0 cm path length = 40 ng/μl at 260 nm wavelength.

### Contamination of mRNA

Two strategies were followed for minimization of contamination of samples by adherent extracellular RNA from other cells ruptured during dissection, a potential problem in RNA collection from microdissected heterogeneous tissues [35]. Multiple rinses of tissues were followed by acute incubation of samples. The effectiveness of acute incubation on degradation of adherent extracellular RNA was tested. Total RNA from rat kidney (7926 Ambion, Austin, TX) was incubated in vials and stored at either 4°C or 37°C for 24 hours. The quality of RNA was subsequently evaluated by Agilent BioAnalyzer. Fig. S1 (See Additional File 1: Fig. S1) shows marked degradation of RNA in the sample incubated at 37°C, supporting the efficacy of this treatment in preventing or reducing potential contamination from adjacent tissues during dissection.

### Quantitative real-time RT-PCR

Primers for TRPV5, TRPV6, calbindin-D9K, calbindin-D28K, NCX1, NCX2, NCX3, PMCA1, PMCA2, PMCA3, PMCA4, vitamin D receptor and 18S were used as described previously [5] and real time RT-PCR was performed by the same procedure and analyzed as described [26]. All PCR reactions were carried out in a final volume of 25 μl in a real-time thermocycler (Smart Cycler, Cepheid, Sunnyvale, CA). The reaction mix consisted of Master Mix (OneStep RT-PCR kit;210210, Qiagen), 0.2× SYBR green 1 (# S7567, Molecular Probes), and 600 nM of each gene-specific forward and reverse primer. Reverse transcription was performed for 30 min at 50°C followed by 15 min at 95°C. PCR conditions used were: denature at 95°C for 60 s, annealing at 51°C - 60°C for 60 s with an extension at 72°C for 60 s, amplified for 50 cycles. Hot-measurements that are 3-5°C below product melting temperature were performed to eliminate signal contamination by primer-dimers [26]. To exclude the possibility of genomic DNA amplification during the PCR reaction, RT-negative controls were performed. PCR products were run on 2% agarose gels and detected by ethidium bromide or with the Bioanalyzer 2100 with DNA 1000 Assay Kit (5065-4449, Agilent Technology). All PCR products displayed a specific band on agarose gel or a specific peak on the Bioanalyzer electropherogram. PCR products were purified (28104, Qiagen) and the purified products were sequenced to verify the identity of the RT-PCR products. The expression level of genes was evaluated by quantitative real-time RT-PCR using 0.5 - 5 ng total RNA per reaction. Transcripts of 18S rRNA and target genes were amplified as described using gene-specific primers. The specific gene expression was normalized to the level of 18S in each sample as described previously [26], taking into account the fidelity of each PCR reaction.

An example of real time RT-PCR results for native SCCD (See Additional File 1: Figure S1) shows cycle-fluorescence growth curves of 18S (dashed line), TRPV5 of control (dotted line) and 1,25-(OH)_2_vitamin D_3 _treated epithelia (solid line). Cycles at threshold = 30 are about 16 for 18S, 32 for TRPV5 of control and 30 for TRPV5 of 1,25-(OH)_2_vitamin D_3 _treated, demonstrating an increase in expression of TRPV5 transcripts after 1,25-(OH)_2_vitamin D_3 _treatment.

### Immunoblots

Western blots were prepared by methods similar to those described previously [36]. Primary cultures of SCCD were used in order to obtain sufficient protein for analysis (see above). SCCD monolayers from three Snapwell inserts for each experimental condition were washed three times in phosphate buffered saline (PBS; 150 mM NaCl, 8 mM Na_2_HPO_4_·2H_2_O, 2 mM KH_2_PO_4_, pH 7.4) at 37°C and then lysed in cold (4°C) RIPA buffer (10 mM Tris base, 1% sodium deoxycholate, 1% Tergitol (NP40S, Sigma St Louis, MO), 150 mM NaCl, pH 7.9) containing 1% protease inhibitor cocktail (P2714, Sigma). Rat kidney cortex and papilla whole cell lysates were obtained from young (3 week) Wistar rats. Tissue was frozen in liquid nitrogen, crushed and homogenized using a mortar and pestle in cold (4°C) RIPA buffer containing protease inhibitors and used as controls. The supernatant was collected after being stored at 4°C for one hour, centrifuged at 15,000 g for 5 min at 4°C; the protein concentration was determined with a Nanodrop ND-1000 Spectrophotometer using the conversion of 1 absorbance unit at 280 nm wavelength = 1 mg protein/ml and stored at -80°C for later use.

Total SCCD protein amounts were prepared to be equal for each gel lane. After being diluted in 1× Laemmli sample buffer (#161-0737, Bio-Rad, Hercules, CA) containing 5% 2-mercaptoethanol (#M-7154, Sigma), samples were heated for 15 min at 75°C. Proteins were separated using 4-15% Tris-HCl precast polyacrylamide gels (#161-1104, Bio-Rad) at 140 V for 60 min using running buffer [25 mM Tris-Base, 192 mM glycine and 0.1% SDS](#161-0732, Bio-Rad). A colored marker mixture (#161-0375, Bio-Rad) was used for estimation of molecular weight. After electrophoresis, proteins were transferred onto a PVDF membrane (#162-0175, Bio-Rad) using the XCell II Blot Module semi-wet transfer unit (#EI0002, Invitrogen) at 24 V for 75 min using transfer buffer [25 mM Tris-Base, 192 mM glycine, 20% methanol, pH 8.3]). The membrane was then blocked with a blocking buffer (5% non-fat dry milk [#170-6404, Bio-Rad] in TBS [20 mM Tris-Base, 137 mM NaCl, pH 7.6] containing 0.05% Tween 20) for 1 hr at room temperature and then probed overnight at 4°C with 1:400 primary antibody (TRPV5, #CAT21A, Alpha Diagnostics, San Antonio, TX). For standardization, membranes were stripped using Restore Western Blot Stripping Buffer (#21059, Pierce) and then reprobed with anti-actin antibody (1:1000, #A2066 Sigma) for 1 hr at room temperature. Horseradish peroxidase-conjugated secondary donkey anti-rabbit IgG (#NA934V Amersham Biosciences, Piscataway, NJ) was diluted to 1:5,000 in blocking buffer and then used to incubate the membrane for 1 hr at room temperature. Antigenic peptide for TRPV5 (#CAT21P, Alpha Diagnostics, 1: 36) was used to validate antibody specificity. Finally, membranes were treated with a chemiluminescent substrate (SuperSignal West Pico Substrate #34080 for actin & SuperSignal West Femto Maximum Sensitivity Substrate #34095 for TRPV5, Pierce, Rockford, IL) and digitally imaged with a Kodak IS4000R image station.

The intensity of each band was analyzed using Molecular Imaging Software version 4.0.5 (Kodak, Rochester, NY). The intensity of signals was obtained by dividing TRPV5 signals by corresponding actin signals. To compare the expression of TRPV5 in the presence and absence of 1,25-(OH)_2_vitamin D_3 _among different membranes, the average intensity of control (absence of 1,25-(OH)_2_vitamin D_3_) bands at each molecular mass on each membrane was normalized to 1.

### Glycosylation

SCCD protein samples were treated with Peptide: N-Glycosidase F (PNGase F) following the manufacturer's instruction (#P0704S; New England Biolabs, Ipswich, MA). Equal amounts of lysates were denatured at 100°C for 10 min in the presence of glycoprotein denaturing buffer. After adding reaction buffer, 10% NP40 and 3 μl of PNGase F or water (for control reaction), samples were incubated at 37°C for 2 hr. Samples were then analyzed by Western blot as described above.

### Immunohistochemistry

Animals (38-45 day old rats) were deeply anesthetized with 4% tribromoethanol (0.016 ml/g body weight i.p.) and transcardially perfused with Cl- free solution (in mM: 150 Na-gluconate, 1.6 K2HPO4, 0.4 KH2PO4, 4 Ca-gluconate, 1 MgSO4 and 5 glucose, pH 7.4) and then with 4% paraformaldehyde in Cl- free solution. The temporal bones were dissected. For whole temporal bone cryosections, temporal bones were post fixed in 4% paraformaldehyde, decalcified for 24-72 hours in 10% (by weight) EDTA in PBS, processed through 10%-30% sucrose gradient and incubated in 30% sucrose in PBS. The temporal bones were embedded in TBS Tissue Freezing Medium (#H-TFM, Triangle Biomedical Sciences, Durham, NC), cryosectioned at 12 μm thickness and mounted on ProbeON Plus charged glass slides (#15-188-52, Fisher).

The slides were warmed for 3 hours at 37°C after cryosectioning and then rehydrated with PBS. The tissues were then permeabilized and blocked using the blocking solution (PBS-TX: 0.2% Triton X-100, 5% BSA in PBS) for 30 min. The sections were incubated overnight at 4°C with the primary antibody (rabbit anti-Trpv5 antibody [#CAT21A] or rabbit anti-Trpv6 antibody [#CAT11A], 1:100, Alpha Diagnostics, San Antonio, TX) with or without blocking peptide ([#CAT21A] and [#CAT11A], 1:4) in 0.2% PBS-TX with 2% BSA. The sections were washed with PBS-TX and then incubated in the dark with the secondary antibody (donkey anti-rabbit Alexa 488 antibody, 1:1000, Molecular Probes) for 1 hour at room temperature. Actin filaments were visualized by staining with Alexa 594 conjugated phalloidin (1:40, Molecular Probes). Finally, the sections were washed with PBS-TX and overlaid with FluorSave (Calbiochem, La Jolla, CA) and a cover glass. Sections were observed with a confocal microscope (LSM510 Meta, Carl Zeiss, Germany).

### Statistics

Data are presented as mean values ± SE from n observations. Student's t-test was used to determine statistical significance after the gene expression ratios were first subjected to a logarithmic transformation to normalize the distribution. Paired or unpaired tests were performed as appropriate for the specific data sets. Differences between means were considered significant for P < 0.05.

## Authors' contributions

DY and KN carried out the study, including microdissection of tissues, primer design and validation, RNA isolation, quantitative analyses of PCR, protein isolation, all steps in the immunoblots and their analysis, preparation of ears for immunohistochemistry and contributed to writing the manuscript. NNR carried out many of the RT-PCR reactions and primer validation studies. DGH performed many of the microdissections and primary cell cultures. RS contributed development of the immunoblot method. PW directed the confocal microscopy aspect of the study, obtained the confocal images and contributed valuable comments and discussion. DCM conceived of the study, and participated in its design and coordination and contributed to writing the manuscript. All authors read and approved the final manuscript.

## Supplementary Material

Additional file 1 **Fig. S1**. Quality and degradation of extracellular RNA and representative qRT-PCR. Click here for file
