# Supplementary material for: Expression of epithelial calcium transport system in rat cochlea and vestibular labyrinth
Source: BMC Physiol. 2010 Jan 29;10:1. doi: 10.1186/1472-6793-10-1 (PMC2825184; doi:10.1186/1472-6793-10-1)
Supplement: Additional file 1 — Fig. S1. Quality and degradation of extracellular RNA and representative qRT-PCR. [file 1472-6793-10-1-S1.PDF]

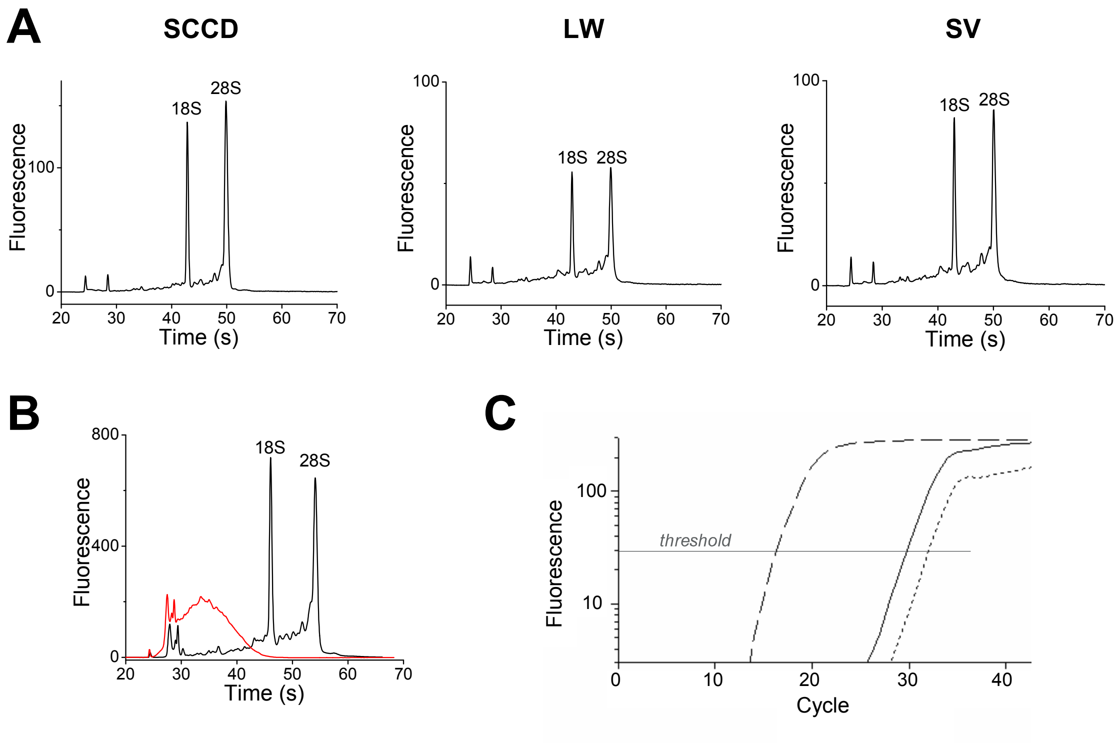

Figure S1 - Quality of total RNA, degradation of extracellular RNA and representative qRT-PCR.

A) Total RNA of native semicircular canal duct (SCCD), lateral wall (LW) and stria vascularis (SV) were evaluated by Agilent 2100 Bioanalyser. The two large peaks (18S and 28S) and low background level clearly show their high quality. Fluorescence is plotted against elution time, which correlates with nucleotide size.

B) Comparison of quality of total rat kidney RNA (initial concentration 10 ng/µl) after 24 hours incubation at 4 ˚C (black line) and 37 ˚C (red line); the latter was strongly degraded and lacked distinct peaks for ribosomal RNA.

C) Representative quantitative RT-PCR of native SCCD for 18S (dashed line) and TRPV5 (control, dotted line; 1,25-$(OH)_2$vitamin D_3 treated, solid line). Threshold (Ct) taken in the quasilinear region of the growth curve. Lower Ct value for vitamin D treatment demonstrates greater expression of TRPV5.
